# Sex is a moderator of the association between *NOS1AP* sequence variants and QTc in two long QT syndrome founder populations: a pedigree-based measured genotype association analysis

**DOI:** 10.1186/s12881-017-0435-2

**Published:** 2017-07-18

**Authors:** Annika Winbo, Eva-Lena Stattin, Ida Maria Westin, Anna Norberg, Johan Persson, Steen M. Jensen, Annika Rydberg

**Affiliations:** 1 Department of Clinical Sciences, Pediatrics, Umeå University, 90187 Umeå, Sweden; 2 Department of Physiology, University of Auckland, Auckland, New Zealand; 3 Department of Immunology, Genetics and Pathology, Science for Life Laboratory, Uppsala University, Uppsala, Sweden; 4 Department of Medical Biosciences, Medical and Clinical Genetics, Umeå University, Umeå, 90185 Sweden; 5 Department of Public Health and Clinical Medicine, Heart Centre, Umeå University, Umeå, 90185 Sweden

**Keywords:** Long QT syndrome, Sequence variants, Sex, Risk stratification, Modifier genes, QT prolongation, Sex-sequence-variant interaction, Genotype-phenotype interactions, Founder populations

## Abstract

**Background:**

Sequence variants in the *NOS1AP* gene have repeatedly been reported to influence QTc, albeit with moderate effect sizes. In the long QT syndrome (LQTS), this may contribute to the substantial QTc variance seen among carriers of identical pathogenic sequence variants. Here we assess three non-coding *NOS1AP* sequence variants, chosen for their previously reported strong association with QTc in normal and LQTS populations, for association with QTc in two Swedish LQT1 founder populations.

**Methods:**

This study included 312 individuals (58% females) from two LQT1 founder populations, whereof 227 genotype positive segregating either Y111C (*n* = 148) or R518* (*n* = 79) pathogenic sequence variants in the *KCNQ1* gene, and 85 genotype negatives. All were genotyped for *NOS1AP* sequence variants rs12143842, rs16847548 and rs4657139, and tested for association with QTc length (effect size presented as mean difference between derived and wildtype, in ms), using a pedigree-based measured genotype association analysis. Mean QTc was obtained by repeated manual measurement (preferably in lead II) by one observer using coded 50 mm/s standard 12-lead ECGs.

**Results:**

A substantial variance in mean QTc was seen in genotype positives 476 ± 36 ms (Y111C 483 ± 34 ms; R518* 462 ± 34 ms) and genotype negatives 433 ± 24 ms. Female sex was significantly associated with QTc prolongation in all genotype groups (*p* < 0.001). In a multivariable analysis including the entire study population and adjusted for *KCNQ1* genotype, sex and age, *NOS1AP* sequence variants rs12143842 and rs16847548 (but not rs4657139) were significantly associated with QT prolongation, +18 ms (*p* = 0.0007) and +17 ms (*p* = 0.006), respectively. Significant sex-interactions were detected for both sequent variants (interaction term *r* = 0.892, *p* < 0.001 and *r* = 0.944, *p* < 0.001, respectively). Notably, across the genotype groups, when stratified by sex neither rs12143842 nor rs16847548 were significantly associated with QTc in females (both *p* = 0.16) while in males, a prolongation of +19 ms and +8 ms (*p* = 0.002 and *p* = 0.02) was seen in multivariable analysis, explaining up to 23% of QTc variance in all males.

**Conclusions:**

Sex was identified as a moderator of the association between *NOS1AP* sequence variants and QTc in two LQT1 founder populations. This finding may contribute to QTc sex differences and affect the usefulness of *NOS1AP* as a marker for clinical risk stratification in LQTS.

## Background

The congenital long QT syndrome (LQTS) is a heterogeneous arrhythmia disorder associated with variable QT prolongation and sudden cardiac death risk, caused by pathogenic sequence variants in genes encoding ion channel proteins involved in cardiac repolarization [1]. The most common LQTS subtype, LQT1, is caused by loss-of-function mutations in the *KCNQ1* gene, responsible for the slow rectifier potassium current, IK_s_, that stabilizes heart rhythm during sympathetic catecholamine release and increased heart rates [2]. The task of patient risk stratification remains a major challenge for clinicians managing LQTS families. In addition to the affected gene and specific pathogenic sequence variant, QT prolongation and arrhythmia risk in LQTS may be modulated by several patient-specific factors, such as sex, age, concurrent use of medications, as well as carriership of common genetic sequence variants, also known as genetic modifiers [3]. Among the genetic modifiers, the nitric oxide synthase 1 adaptor protein (*NOS1AP*) gene, also known as the carboxyl- terminal PDZ ligand of neuronal nitric oxide synthase (*CAPON*) [4] gene, has emerged as a significant genetic marker of QT interval prolongation and arrhythmia risk in the general population [5–12], as well as in LQTS [13, 14]. The reported effect sizes on QT interval associated with *NOS1AP* sequence variants range between 3.5 ms per derived (minor) allele in the general population [15], to 7–8 ms in LQTS [14], and *NOS1AP* genotyping has been proposed as an additional factor to aid in clinical LQTS risk stratification [14].

In the context of genetic modifiers, LQTS founder populations sharing mutations identical by descent constitute excellent research models, as the variability secondary to the disease-causing variant may be controlled for, and the background genetic noise is reduced [16]. In Sweden, two LQT1 founder mutations, *KCNQ1* Y111C [17, 18] and *KCNQ1* R518* [19], with an estimated population prevalence of 1:4000 to 1:7000 and 1:2000 to 1:4000 [17, 19, 20], respectively, account for >25% of index cases with molecularly defined LQTS [21].

While the early detection and primary prophylactic treatment facilitated by our established LQTS family clinic, using active cascade-screening and subsequent clinical management, largely prevents studies of LQTS natural history (symptoms), we here take advantage of the size and relative genetic homogeneity of the LQT1 founder populations in order to perform association testing between heart rate corrected QT interval (QTc) and three non-coding *NOS1AP* sequence variants, chosen for their strong association with QTc in the general population (rs12143842 [9]) and in LQTS populations (rs16847548 and rs4657139 [13, 14]). Taken together, our findings indicate that sex may be a significant moderator of the association between *NOS1AP* sequence variants and QTc prolongation, i.e. conferring different levels of QT prolongation for males and females carrying the same modifier, at least within the context of these LQT1 founder populations.

## Methods

The study sample consisted of 312 individuals (180 females) from two Swedish LQT1 founder populations, including 85 genotype negative individuals (47 females), and 227 genotype positive individuals (133 females), segregating either *KCNQ1* Y111C [17, 18] (*n* = 148, 84 females) or *KCNQ1* R518* [19] (*n* = 79, 49 females). All participants signed an informed consent and the study was approved by the Regional Ethical Committee in Umeå, Umeå University, Sweden (Dnr 05 − 127 M).

The founder status of the two LQT1 populations has been confirmed by both genealogical studies and microsatellite marker analysis [17, 19]. Genotype negative/positive was defined based on carrier status of the familial pathogenic sequence variant, as ascertained by molecular genetic testing (non-carrier/carrier). Individuals carrying more than one *KCNQ1* pathogenic sequence variant were not included in the study. As previously described [18, 19], diagnostic molecular genetic testing in the LQT1 founder populations was performed according to clinical praxis, extracting genomic deoxyribonucleic acids by a standard salting-out procedure and genotypes ascertained in index cases by either targeted mutation analysis of the Swedish founder variants Y111C and R518* using MGB-probes analyzed on ABI 7000 (Applied Biosystems, Foster City, CA, USA) or by screening all coding exons of *KCNQ1* by denaturing high-performance liquid chromatography (Wave 3500 HT, Transgenomic, Inc., Omaha, Neb) and/or Sanger sequencing (CEQ 8000, Beckman Coulter, Fullerton, CA, USA). In family members, carrier status was ascertained by Sanger sequencing (CEQ 8000, Beckman Coulter or ABI 3500xL Dx Genetic Analyzer, Applied Biosystems) or targeted mutation analysis of the identified pathogenic sequence variants using MGB-probes analyzed on ABI 7000 (Applied Biosystems, Foster City, CA, USA).

### Analysis of *NOS1AP* sequence variants

All study participants were genotyped for three previously published sequence variants located in the 5′ upstream regulatory region of the *NOS1AP* gene; rs12143842, rs16847548 and rs4657139, using MGB-probes analyzed on ABI 7000 (Applied Biosystems, Foster City, CA, USA). The rs-numbers for denoting the sequence variants were obtained from the dbSNP database (www.ncbi.nlm.nih.gov/projects/SNP). Ancestral (major) and derived (minor) alleles were denoted as “A” and “a”, respectively (AA- major homozygote, Aa- heterozygote, aa- minor homozygote).

### Electrocardiographic measurements

QT interval duration was measured on 50 mm/s standard 12- lead electrocardiograms, obtained from medical records, recorded in absence of beta-blocker therapy, when available. Electrocardiograms were duplicated, coded and assessed on two separate occasions by one experienced observer. QT intervals were measured manually, preferably in lead II as a mean of at least three consecutive QT intervals, and corrected for heart rate by Bazett’s formula (QT/√R-R), using the mean of the R-R intervals preceding the measured beats. The mean of the two QTc values was used in the subsequent analyses.

### Statistical analyses

Data were summarized and presented as total number plus percentage for proportions, and mean ± standard deviation for continuous variables. Pearson correlations (x^2^ test) were calculated between continuous variables/covariates and results presented with correlation coefficient (r) and associated *p*-value. For the repeated QTc measurements, the mean difference between the two measurements (expressed as mean ± standard deviation, median) and the intra-observer coefficient of variation (standard deviation of the mean/ mean standard error × 100) was calculated and expressed in percent. For association analyses between *NOS1AP* sequence variants and QTc, a minor allele frequency of >0.1 for each *NOS1AP* sequence variant was set as a cut-off for inclusion in association analysis, to limit the risk of a type 1 error. *NOS1AP* genotypes were coded as AA = 0, Aa = 1 and aa = 2 (additive model) and AA = 0, Aa or aa = 1 (dominant model). The effect of *NOS1AP* sequence variants on *KCNQ1* QTc was assessed by linear regression, using a dominant model and results presented with correlation coefficient (r) and unstandardized beta coefficients presented with associated standard error and associated *p*-value. As sex differences were apparent from visual inspection of the data, a multiplicative interaction term between *NOS1AP* genotype and sex was calculated and assessed by x^2^ test, and separate sex-stratified linear regression analyses performed. A priori power calculations stated that at least 223 participants would have to be included in order to be able to detect a difference in QTc of 6 ms or more (statistical power 80%, alpha 0.05), using QTc means and standard deviations from our previously published clinical founder population data [18, 19]. Consequently, the entire study population, and the subgroup genotype positives, satisfied this criterion. For the sex stratified analyses, the study was powered to detect differences in QTc >7 ms (females) and >8 ms (males).

Due to the non-independence among family members in the two included founder populations [22], multivariable analyses of variance were performed using pedigree-based measured genotype association analysis. As previously described [23], relatedness data for the measured genotype association analysis, i.e. identity by descent pedigrees, were constructed by a best estimate approach using previously published genealogical and microsatellite data for all included families [17, 19]. The measured genotype approach [24, 25], estimates genotype-specific trait means in large pedigrees by a fixed-effects model. To control for effects of multiple covariates an initial maximum likelihood model was constructed for the primary trait QTc and covariates *NOS1AP* genotype (dominant model, AA = 0, Aa and aa = 1), *KCNQ1* genotype (positive/negative), sex, and age at electrocardiogram, as well as interaction terms between sex and included covariates. Sex stratified analyses included the covariates *NOS1AP* genotype, *KCNQ1* genotype and age at electrocardiogram. For each analysis, a final restricted model was constructed comprising covariates with a *p* value <0.1. For the final models, the polygenic heritability and associated *p*-value (H2r: corresponding to the proportion of the phenotype variance in a trait that is attributable to the additive effects of genes), the proportion of variance caused by the covariates, and the residual kurtosis (within normal range if <0.8) were calculated. For analyses with a residual kurtosis >0.8 the tdist command was used to normalize data. For all covariates included in the final model, beta coefficients (corresponding to the effect size on QTc predicted by the model) were calculated.

For all analyses, a two-tailed *p* value of <0.05 was considered statistically significant. Statistical analyses were performed with SPSS 23.0 (Statistical Package for Social Sciences, SPSS Inc., Chicago, Ill) and Sequential Oligogenic Linkage Analysis Routines software (SOLAR) [26] available at http://solar-eclipse-genetics.org.

## Results

### QTc variability in two LQT1 founder populations

An overview of the study sample, with regards to genotype, sex, age at the electrocardiographic recording and mean QTc, is summarized in Table 1. Electrocardiograms were available for all 312 participants (227 genotype positive and 85 genotype negative). For the repeated QTc measurements, the mean difference between the two measurements was 2.7 ± 9 ms, median 2.0 ms, standard error 0.5, with an intra-observer coefficient of variation of 0.17%. A substantial variance in mean QTc was seen throughout the study population; all participants 464 ± 38 ms, genotype positive 476 ± 36 ms (Y111C 483 ± 34 ms; R518* 462 ± 34 ms) and genotype negative 433 ± 24 ms (Table 1). As expected, female sex correlated with longer QTc in all genotype groups (all *r* = 0.238, *p* < 0.001, genotype positive *r* = 0.251, *p* < 0.001, genotype negative *r* = 0.303, *p* = 0.005), with effect sizes, as calculated by linear regression, ranging from +14 ms (Y111C genotype positive), +15 ms (genotype negatives) to +18 ms (all genotype positives) to +30 ms (R518* genotype positive), as compared to the males of the same genotype.

**Table 1:** Overview of the Swedish LQT1 founder study population

	N (% females)	Age at ECG (years)	QTc (ms)
All participants	312 (58)	31 ± 22	464 ± 38
Genotype positive	227 (59)	31 ± 21	476 ± 36
*KCNQ1* Y111C	148 (57)	32 ± 22	483 ± 34
*KCNQ1* R518*	79 (62)	29 ± 19	462 ± 34
Genotype negative	85 (55)	33 ± 23	433 ± 24

### Single covariate analyses for NOS1AP sequence variants and QTc

All three studied *NOS1AP* sequence variants had a minor allele frequency > 0.1 (0.3–0.4) in the study population (Table 2). Two of the three studied *NOS1AP* sequence variants (rs12143842 and rs16847548, in a strong and significant linkage, *r* = 0.882, *p* < 0.001) showed significant association with QTc prolongation in the study population, whereas rs4657139 did not. For rs12143842 and rs16847548, carriership of an increasing number of derived alleles was positively correlated with QTc prolongation in the study population (*r* = 0.113, *p* = 0.046, and *r* = 0.124, *p* = 0.028, respectively). By single covariate regression analysis, carriership of one or more derived alleles was associated with a + 12 ms (rs12143842, *p* = 0.01) and +10 ms (rs16847548, *p* = 0.02) QTc prolongation, respectively, in genotype positives. Due to the strong linkage between rs12143842 and rs16847548, stratified analyses for *KCNQ1* genotype, (genotype positives, Y111C carriers, R518* carriers and genotype negatives), were performed for the *NOS1AP* sequence variant with the strongest association to QTc prolongation, rs12143842, Table 3. In summary, in the study population carriership of one or more derived rs12143842 alleles was associated with moderate QTc prolongation, either significant or approaching significance, across all genotype groups (+9 ms to +13 ms, top third of Table 3).

**Table 2:** Allele frequencies for three non-coding sequence variants in the upstream 5′ regulatory region of the *NOS1AP* gene, analysed in two LQT1 founder populations

Sequence variant	Alleles (A/a)	Genotype	N (%)	MAF
rs12143842	C/T	CC	150 (48)	0.3
		CT	135 (43)	
		TT	27 (9)	
rs16847548	T/C	TT	154 (49)	0.3
		TC	137 (44)	
		CC	20 (6)	
rs4657139	T/A	TT	124 (40)	0.4
		TA	148 (47)	
		AA	39 (13)	

*A* ancestral or major allele, *a* derived or minor allele, *MAF* minor allele frequency

**Table 3:** Association testing between *NOS1AP* rs12143842 and QTc, stratified by genotype and sex, in two LQT1 founder populations

Study population	N	AA N	AA QTc^a^	Aa/aa N	Aa/aa QTc^a^	Effect^b^	P^c^
All participants	312	150	458 ± 35	162	469 ± 40	+11 (4)	**0.011**
All genotype positive	227	109	470 ± 33	118	481 ± 37	+12 (5)	**0.013**
All Y111C	148	73	476 ± 29	75	489 ± 33	+13 (6)	**0.014**
All R518^a^	79	36	457 ± 36	43	467 ± 32	+10 (8)	0.211
All genotype negative	85	41	428 ± 23	44	437 ± 25	+9 (5)	0.090
All females	180	82	470 ± 32	98	473 ± 37	+4 (5)	0.458
Genotype positive females	133	62	480 ± 28	71	486 ± 31	+6 (5)	0.244
Y111C females	84	39	484 ± 25	45	493 ± 28	+9 (6)	0.122
R518^a^ females	49	23	473 ± 34	26	474 ± 35	+1 (10)	0.926
Genotype negative females	47	20	437 ± 21	27	440 ± 28	+3 (7)	0.702
All males	132	68	445 ± 35	64	463 ± 43	+18 (7)	**0.009**
Genotype positive males	94	47	456 ± 33	47	474 ± 44	+18 (8)	**0.028**
Y111C males	64	34	467 ± 31	30	485 ± 49	+18 (10)	0.080
R518^a^ males	30	13	428 ± 23	17	456 ± 26	+27 (9)	**0.007**
Genotype negative males	38	21	419 ± 21	17	431 ± 18	+12 (7)	0.060

AA- ancestral rs12143842 genotype, Aa/aa- carrier of one or two derived/minor rs12143842 alleles, respectively

^a^Manually measured on 12-lead resting electrocardiograms and heart rate corrected using Bazett’s formula, in ms

^b^The effect on QTc, in ms, if having one or more rs12143842 minor alleles (a), as compared to ancestral genotype (AA), i.e. unstandardized beta coefficient, followed by standard error in parenthesis

^c^As calculated by linear single covariate regression analysis (in bold when significant at the <0.05 level)

### Sex as a moderator of the association between *NOS1AP* sequence variants and QTc

Based on visual inspection of the data, sex interaction effects were suspected. A multiplicative interaction term for sex and rs12143842 was calculated, revealing a significant sex-sequence variant interaction, *r* = 0.892, *p* < 0.001 (rs16847548 *r* = 0.944, *p* < 0.001).

Notably, when performing sex stratified analyses, there was no correlation between the number of rs12143842 derived alleles carried and QTc prolongation in females (AA = 470 ± 32, Aa = 477 ± 36, aa = 451 ± 30, *r* = −0.031, *p* = 0.7), while in males the expected pattern of increasing QTc with a higher burden of derived alleles was seen (AA = 445 ± 35, Aa = 459 ± 35, aa = 479 ± 65, *r* = 0.265, *p* = 0.02). In genotype-positive participants the corresponding results were *r* = 0.007, *p* = 0.9 for females, and *r* = 0.262, *p* = 0.01 for males, and similar trends were seen in genotype-negatives.

Using a dominant model, the above finding was reproduced across all *KCNQ1* genotype groups, as presented in the bottom two thirds of Table 3, detailing the results of the sex- and genotype stratified single covariate regression analyses for rs12143842 and QTc length in the LQT1 founder populations. Carriership of one or more derived rs12143842 alleles showed no significant association with QTc length in founder population females irrespective of *KCNQ1* genotype, while a significant QTc prolongation between +18 ms to +27 ms was seen in males (all males, genotype positive males, and R518* males), and similar trends were present in Y111C males and genotype negative males (Table 3).

A QTc >500 ms was noted for 24/180 (13%) of females and 9/132 (7%) of males. To test whether the higher prevalence of marked QTc prolongation in females was a confounding factor (i.e. skewing QTc means), we performed correlation analyses between rs12143842 and QTc excluding participants with a QTc >500 ms. This did not improve the non-significant correlation between rs12143842 and QTc in females, but strengthened the correlation between rs12143842 and QTc for males (correlation *r* = 0.268, *p* = 0.003, effect size +14 ms, *p* = 0.003 in the regression analysis).

### Multivariable measured genotype association analyses in the LQT1 founder populations

Due to the founder status of the included populations, the association between *NOS1AP* genotype and QTc prolongation in the study population was further analysed by measured genotype association analysis, adjusting for relatedness in the LQT1 founder populations, as well as covariates *KCNQ1* genotype, sex, age at electrocardiogram, and sex-covariate interactions. As expected, QTc as a trait demonstrated significant heritability, with a heritability measure (H2r) of 0.48, *p* < 0.0001. Separate associations between QTc prolongation and rs12143842 (+18 ms, *p* = 0.0007) and rs16847548 (+17 ms, *p* = 0.006) were verified by multivariable measured genotype association analysis, correcting for relatedness, *KCNQ1* genotype, sex, sex*covariate interaction and age at the electrocardiographic recording. No significant association was seen for rs4657139. As rs12143842 and rs16847548 showed significant linkage (*r* = 0.882, *p* < 0.001), the *NOS1AP* sequence variant with the strongest association to QTc prolongation in the founder populations, rs12143842, was included in the final model (Table 4). Taken together, all significant covariates in the final relatedness-adjusted model (*KCNQ1* genotype, rs12143842 genotype, sex and sex*rs1214382 interaction) explained 31% of QTc variance in the study population (Table 4).

**Table 4:** Final restricted maximum likelihood model, using pedigree-based measured genotype association, explaining variance in QTc in the LQT1 founder populations

Covariates^a^	Beta	SE	*P*-value^b^
*In the entire study population (n = 312)*			
*KCNQ1* genotype (positive)	+47	3.9	2.0 × 10^−13^
rs12143842 (Aa or aa)	+18	5.4	0.0007
Sex (female)	+23	4.9	0.004
Sex*rs12143842 interaction	-12	6.7	0.08
Proportion of variance explained			32%
*In females (n = 180)*			
*KCNQ1* genotype (positive)	+44	4.9	6.4 × 10^−17^
Age at electrocardiogram (per year)	+0.22	0.1	0.02
Proportion of variance explained			31%
*In males (n = 132)*			
*KCNQ1* genotype (positive)	+47	6.1	5.6 × 10^−11^
rs12143842 (Aa or aa)	+19	6.0	0.002
Proportion of variance explained			23%

Beta- unstandardized beta coefficient (~effect size, ms), SE- standard error

^a^Inclusion criteria in final model *p* < 0.1 (interactions sex*KCNQ1 genotype and sex*age at electrocardiogram were excluded from all models, rs12143842 was not significantly associated with QTc in females (*p* = 0.16) and excluded from the final model, increasing age at electrocardiogram was significantly associated with a longer QTc in females only)

^b^Calculated by pedigree-based measured genotype association analysis using SOLAR software (solar-eclipse-genetics.org)

Importantly, when performing sex stratified multivariable measured genotype association analyses in the study population (including covariates *NOS1AP* genotype, *KCNQ1* genotype and age at electrocardiogram) and adjusting for relatedness, neither rs12143842 nor rs16847548 were associated with QTc prolongation in females (*p* = 0.16 for either sequence variant), whereby the final multivariable model explaining QT variance included only covariates *KCNQ1* genotype and age at electrocardiogram. In females, increasing age was significantly associated with QTc prolongation, with the model predicting a + 0.22 ms increase in QTc per added year of age, *p* = 0.02, Table 4. In males, there was a significant association between *NOS1AP* sequence variant rs12143842 and QT prolongation (+19 ms, *p* = 0.002). The corresponding results for rs16847548 was +8 ms, *p* = 0.02. In males, the final relatedness-adjusted multivariable model, including *NOS1AP* and *KCNQ1* genotype, but not age at electrocardiogram, explained 23% of QTc variance (20% for rs16847548), Table 4.

## Discussion

We used a pedigree-based measured genotype association analysis to study the association between QTc prolongation and three previously published *NOS1AP* sequence variants, chosen for their strong association with QTc in LQTS and normal populations, in two large Swedish LQT1 founder populations, revealing a significant association for rs12143842 and rs16847548, but not rs4657139. As in the general population [9], the strongest association to QTc in the LQT1 founder populations was seen for rs12143842. Importantly, sex was revealed as a significant moderator of the association between *NOS1AP* sequence variants and QTc in the LQT1 founder populations, across all genotype groups. In a multivariable analysis, correcting for *KCNQ1* genotype, age at electrocardiogram and relatedness, one or more derived rs12143842 alleles predicted a + 19 ms QTc prolongation (*p* = 0.002) in males only, while no significant association with QTc prolongation was seen in females (where *KCNQ1* genotype and age were the only covariates significantly associated with QTc). This is the first study reporting significant sex-*NOS1AP* sequence variant interactions in LQTS, suggesting rs12143842 and rs16847548 might not be sex neutral risk markers.

Sequence variants in the 5′ upstream regulatory region of the *NOS1AP* gene were first identified as significant genetic modifiers of QT duration in a population-based genome wide association study, and remain among the strongest QT modifiers identified in the general population*.* [6, 15] The exact mechanisms and specific causative sequence variant(s) remain to be defined, whereby significantly associated variants are typically linked, such as the rs12143842 and rs16847548 variants in this study.

Mechanistically, *NOS1AP* overexpression shortens repolarization via decreased L-type calcium currents and increased rapid rectifier potassium currents (IK_r_) in isolated ventricular cardiomyocytes [27], providing a rationale for how *NOS1AP* sequence variants could modulate QT interval in humans, and specifically in LQT1, where the dependence on IK_r_ to compensate for LQT1-related IK_s_ reduction is enhanced [28]. *NOS1AP* variants causing loss-of-function *NOS1AP* effects would thus be expected to prolong repolarization, corresponding to an increased QTc in LQT1 and possibly also in genotype negatives as suggested in this study and in previous population studies [9]. Moreover, emerging data from studies on male rats identify *NOS1AP* as an important modifier of sympathetic tone in cardiac neurons, a mechanism that may be of specific importance in LQT1 [29, 30]. However, importantly, so far, all animal studies into *NOS1AP* function seem to have been performed in male animals (standard).

The first association testing regarding *NOS1AP* variants in LQTS patients was performed in carriers of the *KCNQ1* A341V pathogenic variant (*n* = 205) from a South African founder population, reporting a significant association between variants rs4657139 and rs16847548 and the probability of having a QTc in the top 40% (rs4657139, *p* = 0.03; rs16847548, *p* = 0.03) [13]. No significant association between derived *NOS1AP* sequence variants and scalar QTc prolongation was seen, a finding that was interpreted as secondary to the sample size [13]. A larger study based on a prospective LQTS register including 901 genotype positive individuals, reported a significant association between rs4657139 and rs16847548 and QTc (+7 ms, *p* < 0.05 and +8 ms, *p* < 0.01, respectively). None of the previous studies on *NOS1AP* variants in LQTS populations have reported sex stratified data, nor commented on sex specific effects (or lack thereof) [13, 14]. Notably, the average effect size on QTc for the *NOS1AP* sequence variants in the Swedish LQT1 founder populations, without sex-stratification (+11 ms for rs12143842 and +7 ms for rs16847548) is similar to previously published effect sizes [13, 14].

Sex-sequence variant interactions for *NOS1AP* have been reported in the population-based GRAPHIC study (*n* = 919 females, 918 males), where the derived allele of *NOS1AP* rs10494366 significantly prolonged QTc by +5 ms in females only (*p* = 8 × 10^−7^, sex-sequence variant interaction term *p* = 0.025) [8]. Significant male-specific effects on QT duration for genetic modifiers have been reported previously: in Israeli families carriership of the *KCNE1* G38S sequence variant (rs1805127) was associated with a + 22 ms QTc prolongation in males only (*p* = 0.007) [31]; in a First Nations *KCNQ1* founder population the combined genotype L353 L*V250 M was associated with a + 35 ms QTc prolongation in males only (*p* = 0.003) [32]; and in the Finnish *KCNQ1* G589D founder population (*n* = 279 females, 213 males), carriership of the *KCNE1* D85N sequence variant (rs1805128) was associated with a + 26 ms QTc prolongation in males only (*p* = 0.003, sex-sequence variant interaction term *p* = 0.028) [33].

The potential mechanisms underlying this apparent sex-specific moderation of QT prolongation by certain genetic modifiers, in this study *NOS1AP* sequence variants, remains to be elucidated. It is well established that sex influences QTc in an age dependent manner in both LQTS and the general population [34]. This has mainly been attributed to the modulating effects of androgens and oestrogens whose levels naturally differ between the sexes as well as over time. Expression-levels of *KCNQ1* and *KCNH2* have been shown to differ in an age-and-sex dependent manner when studied in independent LQTS families (*n* = 163, whereof 98 LQTS patients), including significantly different mRNA levels in children, adults and adults over the age of 55 years [35]. Interestingly, significantly lower *KCNQ1* and *KCNH2* mRNA levels were detected in male LQTS patients as compared to females (*p* = 0.0084; *p* = 0.035), possibly indicating an increased vulnerability in LQTS males to effects of *NOS1AP* loss-of-function sequence variants affecting repolarization reserve.

With regards to limitations, in the present study, the effects of multiple covariates on QTc, including *NOS1AP* genotype, *KCNQ1* genotype, sex, and age were assessed in the pedigree-based measured genotype association analysis, however, as the study population is relatively young (mean age 31 ± 22 years) and otherwise healthy, the model did not include additional covariates, such as obesity or drug use (medical or other), which may influence QTc. Also, while effects of derived rs12143842 and rs16847548 sequence variants on QTc were significant in males only and effect sizes significantly larger in males as compared to females, the general trend of derived alleles was consequently QT prolonging in all participants of all genotype groups. As the sex stratified analyses were powered to detect effects on QTc >7 ms, the lack of significance for the mean prolonging effect of ~4 ms in females (similar to that seen in the general population [15]) may well be due to sample size, i.e. indicating a moderating effect for sex on effect size per se, rather than an isolated male-specific effect. Still, it is notable that, in this study, across the genotype groups, the significant male effects of *NOS1AP* sequence variants on QTc are approximately double that of the group mean, and more than three times that of the females. Clearly, to formally assess small effects, or to test interactions of sex and sequence variants by age groups, i.e. in relation to possible complex effects secondary to sex hormones throughout life, a larger sample size is needed.

In summary, further studies on possible interactions between sex and sequence variants in basic science models as well as heterogeneous LQTS samples are needed to elucidate underlying mechanisms as well as whether our finding is specific to these founder populations or generalizable to the LQT1/LQTS populations, especially as *NOS1AP* sequence variants are emerging as markers not only for QT prolongation but also clinical risk stratification for LQTS families [13, 14, 36]. In this context, the need for further study of possible sex differences in *NOS1AP* sequence variant effects prior to using these markers for general clinical risk stratification becomes evident*.*

## Conclusions

In this study, sex was identified as a moderator of the association between *NOS1AP* sequence variants and QTc prolongation in two LQT1 founder populations. This finding may be of significance when applying *NOS1AP* genotype to clinical risk stratification, as our data suggests that the effects of this risk marker on QTc are not sex neutral.

## Abbreviations

CAPON: Carboxyl- terminal PDZ ligand of neuronal nitric oxide synthase
IK_r_: Rapid rectifier potassium current (KCNH2-gene encoded)
IK_s_: Slow rectifier potassium current
KCNE1: Gene, encoding beta-subunit of the voltage gated Kv.7.1 channel protein
KCNQ1: Gene, encoding the alfa-subunit of the voltage-gated Kv.7.1 channel protein
LQT1: Long QT syndrome type 1
LQTS: Long QT syndrome
NOS1AP: Nitric oxide synthase 1 adaptor protein gene
